# Bone-stromal cells up-regulate tumourigenic markers in a tumour-stromal 3D model of prostate cancer

**DOI:** 10.1186/1476-4598-12-112

**Published:** 2013-09-30

**Authors:** Louisa CE Windus, Tristan T Glover, Vicky M Avery

**Affiliations:** 1 Discovery Biology, Eskitis Institute for Drug Discovery, Griffith University, Nathan, 4111 Brisbane, QLD, Canada

**Keywords:** Prostate cancer, Tumour-stromal microenvironment, 3D co-cultures, EMT markers, Chemokine CXCR7, Integrins

## Abstract

**Background:**

The cellular and molecular mechanisms that mediate interactions between tumour cells and the surrounding bone stroma are to date largely undetermined in prostate cancer (PCa) progression. The purpose of this study was to evaluate the role of alpha 6 and beta 1 integrin subunits in mediating tumour-stromal interactions.

**Methods:**

Utilising 3D *in vitro* assays we evaluated and compared 1. Monocultures of prostate metastatic PC3, bone stromal derived HS5 and prostate epithelial RWPE-1 cells and 2. Tumour-stromal co-cultures (PC3 + HS5) to ascertain changes in cellular phenotype, function and expression of metastatic markers.

**Results:**

In comparison to 3D monocultures of PC3 or HS5 cells, when cultured together, these cells displayed up-regulated invasive and proliferative qualities, along with altered expression of epithelial-to-mesenchymal and chemokine protein constituents implicated in metastatic dissemination. When co-cultured, HS5 cells were found to re-express N-Cadherin and chemokine receptor CXCR7. Alterations in N-Cadherin expression were found to be mediated by soluble factors secreted by PC3 tumour cells, while chemokine receptor re-expression was dependent on direct cell-cell interactions. We have also shown that integrins beta 1 and alpha 6 play an integral role in maintaining cell homeostasis and mediating expression of E-Cadherin, N-Cadherin and vimentin, in addition to chemokine receptor CXCR7.

**Conclusions:**

Collectively our results suggest that both PC3 and HS5 cells provide a “protective” and reciprocal milieu that promotes tumour growth. As such 3D co-cultures may serve as a more complex and valid biological model in the drug discovery pipeline.

## Introduction

It is well established that the reciprocal interaction of tumour cells with local bone stroma at the metastatic site plays a critical role in metastatic dissemination in prostate cancer (PCa) [1,2]. To-date however, studies have not yet addressed how at the cellular level, these tumour-stromal interactions affect important protein constituents implicated in metastatic dissemination including epithelial-to-mesenchymal transition (EMT) proteins and chemokine receptor expression. Here we have undertaken direct comparisons between 3D monocultures and tumour-stromal co-cultures, temporally comparing their expression of tumourigenic markers. In addition, we have established a role for both β1 and α6 integrin subunits in mediating tumour-stromal interactions.

Recently the evaluation of tumour-stromal cell interactions has been undertaken using a 3D co-culture model. The importance of studying tumours in 3D has been previously described [3,4]. Using these models, studies have shown that when cultured with PCa cells, stromal cells express increased levels of extracellular matrix (versican and tenascin) and chemokine (CCL5, CXCL5, and CXCL16) genes [5], consistent with metastatic clinical tissue samples. Highlighting the reciprocal nature of tumour-stromal interactions, others have shown that when PCa LNCaP cells are co-cultured with human prostate or bone stromal cells in 3D conditions, permanent genetic, morphological and behavioural changes are seen in LNCaP cells [6] indicative of a more invasive phenotype.

An important first step in establishing communication between metastasising cancer cells and surrounding bone stromal cells is the exit of cancer cells from the vasculature once in the bone marrow. Studies suggest that the chemokine, CXCL12, plays a role in trafficking PCa cells to the bone. CXCL12 is expressed by stromal cells in target organs of PCa metastasis (bone, brain, lymph), but not in other tissues [7] and its receptors, CXCR4 and CXCR7, are highly expressed by bone metastatic PCa cells [8,9]. The direct role CXCR7 may play, once PCa cells have established contact with surrounding bone stromal cells, is still unclear. However, growing evidence supports a role for cooperative signalling between integrins and CXCRs in establishing cross-talk between tumour and stromal cells, and colonisation of tumour cells to the bone [10].

Tumour cells localize to bone regions through integrin-mediated contacts with the extracellular matrix (ECM) and stromal cells. Heavily implicated in PCa bone metastases development and progression is the integrin β1 subunit [11-13]. Expression of α5β1 and α2β1 on PCa cells has been reported to facilitate interactions with bone stromal cells [13] and to actively promote invasion and adherence of PCa cells to the bone stroma *in vitro*[12] and experimental bone metastases *in vivo*[11]. Similarly the laminin-binding integrin α6β1 has been shown to permit extravasation of human prostate cancer cells from circulation to the bone stroma *in vivo*[14-16]. While experimental evidence has clearly shown a direct role for integrins α5β1 and α2β1 it is not yet clear how α6β1 may then mediate tumour-stromal interactions once the tumour cells have reached the bone micro-environment. It is the aim of the current paper to further clarify the roles α6 and β1 subunits may have in mediating bone tumour-stromal interactions.

Another important factor that allows PCa cells to infiltrate surrounding tissues and metastasise is the induction of EMT. The common feature of EMT is the loss of E-Cadherin and up-regulation of N-Cadherin and vimentin [17,18]. Evidence of EMT has been provided in both *in vitro* and *in vivo* models [19-21] with the switch believed to initiate release and dissemination of cancer cells from the organ of origin. It has also been suggested that once disseminated, mesenchymal tumour cells recruited to the target organ may undergo a reversal from mesenchymal-to-epithelial transition (MET). Evidence of MET has been limited to *in vitro* and xenograft experiments primarily in breast and bladder cancer [22,23]. From these experiments it has been suggested that MET of the tumour cells may not be driven by cell intrinsic mutations but is under the influence of the pre-metastatic niches in distal organs [24,25]. Surprisingly, few studies have evaluated and validated the occurrence of EMT/MET in *in vivo* prostatic models. To-date one study has confirmed the progressive nature of EMT in prostate cells during xenograft tumour formation and metastasis [26]. Consistent with previous findings in breast cancer, in this prostate model, cancer cells acquire cellular plasticity and EMT progression primarily through interactions with the host tumour micro-environment [26]. Thus in the current study we further evaluated EMT/MET proteins of interest including E-Cadherin, N-Cadherin and vimentin.

Here we evaluate and compare both monocultures and co-cultures of metastatic PC3 cells and bone stromal derived HS5 cells using 3D *in vitro* models. In comparison to monocultures, cells in tumour-stromal co-cultures display alterations in morphology, invasion, proliferation and expression of chemokine and EMT markers. Moreover, mediation of EMT and chemokine markers by α6β1 integrins is altered in co-cultures when compared to their monocultured counterparts. Collectively, our results suggest that stromal cells are extremely plastic and together with metastatic cells can co-operate in a reciprocal manner to produce an emergent behaviour that is more malignant. These results may give further insight into the limitations of specific therapeutics that target tumour cells alone.

## Results

### Characterisation of tumour-stromal co-culture morphology

To investigate differences in morphological characteristics and cell junction formation between HS5, PC3 and tumour-stromal co-cultures (HS5 + PC3 and HS5 + DU145), we used differential inference contrast (DIC) optics, immunostaining and imaging techniques to reconstruct 3D images from cells grown in 3D cultures. The described 3D model consists of cells grown as 3D spheroids following plating on a bed of extracellular matrix, Matrigel. In order to distinguish HS5, DU145 and PC3 cells in co-culture, we used a bone marrow stromal cell specific marker, STRO-1 [27] to visualise HS5 cells. To-date there are no known tumourigenic specific markers for PC3 or DU145 cells, thus to visualise all cells in culture we used a cytoplasmic and nucleic general stain; Cell Mask. We could then determine that cells negative for STRO-1 but positive for Cell Mask were tumour cells, while cells that were both STRO-1 and Cell Mask positive were HS5 cells.

**Figure 1:**
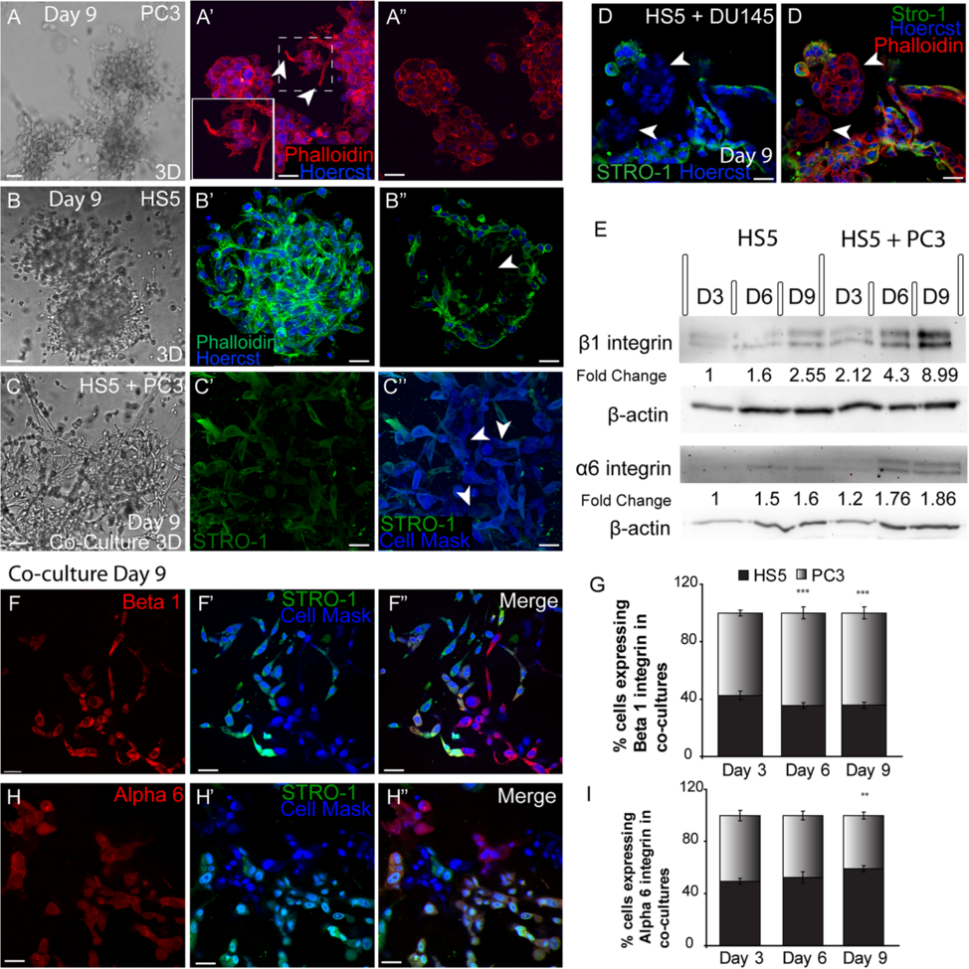
**Morphology of metastatic PC3 and Bone stromal (HS5) cells in monoculture and co-culture (PC3 + HS5) conditions. (A)** Differential Interference Contrast (DIC) images of PC3 cells. **(A’)** F-actin staining of a 3D reconstruction of a PC3 spheroid mass. PC3 cells displayed radiating tubular structures (filled arrowheads). **(A”)** A central Z-slice of a 3D PC3 mass. **(B)** DIC images of HS5 cells. **(B’)** F-actin staining of a 3D reconstruction of a HS5 spheroid mass. **(B”)** A central Z-slice of a 3D HS5 mass displaying an absence of cells in the inner region (arrow). **(C)** DIC images of a co-cultured cells. **(C’)** Immunostaining of co-cultures revealed that HS5 (STRO-1) and **(C”)** PC3 cells (Cell Mask blue; arrows) formed cell-cell contacts. **(D**-**D’)** Immunostaining of co-cultures with HS5 (STRO-1) and DU145 cells (arrows) revealed an absence of cell-cell contacts. **(E)** Western blot and densitometric analysis of endogenous expression of α6 and β1 integrin subunits in monocultured HS5 cells and in co-cultures over 9 days. **(F**-**F’)** Immunostaining of endogenous β1 integrin expression on HS5 (STRO-1 positive; green fluorescence) and PC3 (cell mask blue positive; STRO-1 negative) cells in co-culture. **(G)** Quantification of the percentage of PC3 and HS5 cells expressing β1 integrin in co-culture. **(H**-**H’)** Immunostaining of endogenous α6 integrin expression on HS5 (STRO-1 positive; green fluorescence) and PC3 (cell mask blue positive; STRO-1 negative) cells in co-culture. i) Quantification of the percentage of PC3 and HS5 cells expressing α6 integrin in co-culture (**p < 0.01, ***p < 0.001). Error bars denote S.E.M. Scale bars = 40 μm.

When plated on Matrigel matrix, both stromal and tumour cells clearly differentiated and formed relevant multi-cellular structures. In agreement with our previous findings [4,28], PC3 cells formed irregular shaped clusters (Figure 1A-A”) with stellate radiating tubular processes (Figure 1A’; arrowheads). Consistent with metastatic tumour formation *in vivo*, a central Z-slice of PC3 cells stained for F-actin showed no evidence of polarisation or lumen formation within the centre of the cell mass (Figure 1A”). HS5 stromal cells formed (Figure 1B) rounded masses marked by a meshwork of interlacing cells primarily around the outer regions of the mass (Figure 1B’), with a distinct absence of cells in the inner region (Figure 1B”; arrowhead). These masses clearly lacked cell polarisation and acinar formation.

When co-cultured with PC3 cells, HS5 bone stromal cells (Figure 1C’-C”; green fluorescence) lost their ordered cellular phenotype becoming loosely aggregated, a characteristic associated more readily with an invasive metastatic phenotype. HS5 cells clearly integrated with PC3 cells (Figure 1C”; arrowheads) forming cell-cell contacts. Interestingly, when plated with another PCa metastatic cell line, DU145 cells, HS5 cells retained their characteristic phenotype and rarely formed cell-cell contacts with DU145 cells whose rounded phenotype was maintained in this co-culture (Figure 1D-D’; arrowheads). These results suggest that HS5 cells have a high affinity to interact specifically with bone derived metastatic cells.

### Endogenous protein expression of α6β1 integrin

Previously, we have shown that in comparison to the prostate epithelial cell line RWPE-1, PC3 cells in 3D displayed an up-regulation in the total protein expression of β1 integrin and a down-regulation of α6 integrin expression [4]. Following on from these findings we then wanted to establish whether HS5 and tumour-stromal co-cultures expressed integrin subunits α6 and β1. Densitometric results revealed that similar to expression levels previously reported for prostate epithelial RWPE1 cells [4], HS5 cells expressed minimal levels of β1 integrin with a two fold increase in total protein observed by day 9 in culture (Figure 1E). Consistent with the addition of PC3 cells that are known to express high levels of β1 integrin, tumour-stromal co-cultures displayed a significantly higher level of total β1 integrin protein levels across all days in culture with a 9 fold increase evident by day 9 (Figure 1E). HS5 cells expressed minimal but detectable levels of α6 integrin at days 6 and 9 (Figure 1E). In comparison to HS5 cells, there was a slight up-regulation of α6 integrin expression in co-cultures with a 1.8 fold increase apparent by day 9.

### Beta 1 and α6 integrin expression on HS5 and PC3 cells in co-culture

To further clarify the relative proportion of β1 and α6 integrin expression on the two different cell types in co-culture, immunoassaying was undertaken at days 3, 6 and 9 and script analysis was employed. A similar proportion of HS5 and PC3 cells expressed β1 integrin at day 3, however at day 6 and 9, a significantly higher percentage of PC3 cells were found to express β1 integrin in comparison to HS5 cells (Figure 1G). Immunostaining revealed that in comparison to HS5 cells (Figure 1F’; STRO-1 positive), PC3 cells expressed β1 integrin at higher intensities (Figure 1F-F”). Alternatively, the proportion of cells expressing α6 integrin at days 3 and 6 were similar. By day 9 the percentage of HS5 cells expressing α6 significantly increased in comparison to PC3 cells (Figure 1I). Immunostaining revealed that while the percentage of cells increased, the general intensity of the α6 stain was similar on both PC3 and HS5 cells (Figure 1H-H”).

These results suggest that in tumour-stromal co-cultures, a higher percentage of PC3 cells express β1 at higher intensities while α6 integrin is expressed more consistently by HS5 cells.

### Integrin α6β1 inhibition leads to phenotypic and morphological alterations

**Figure 2:**
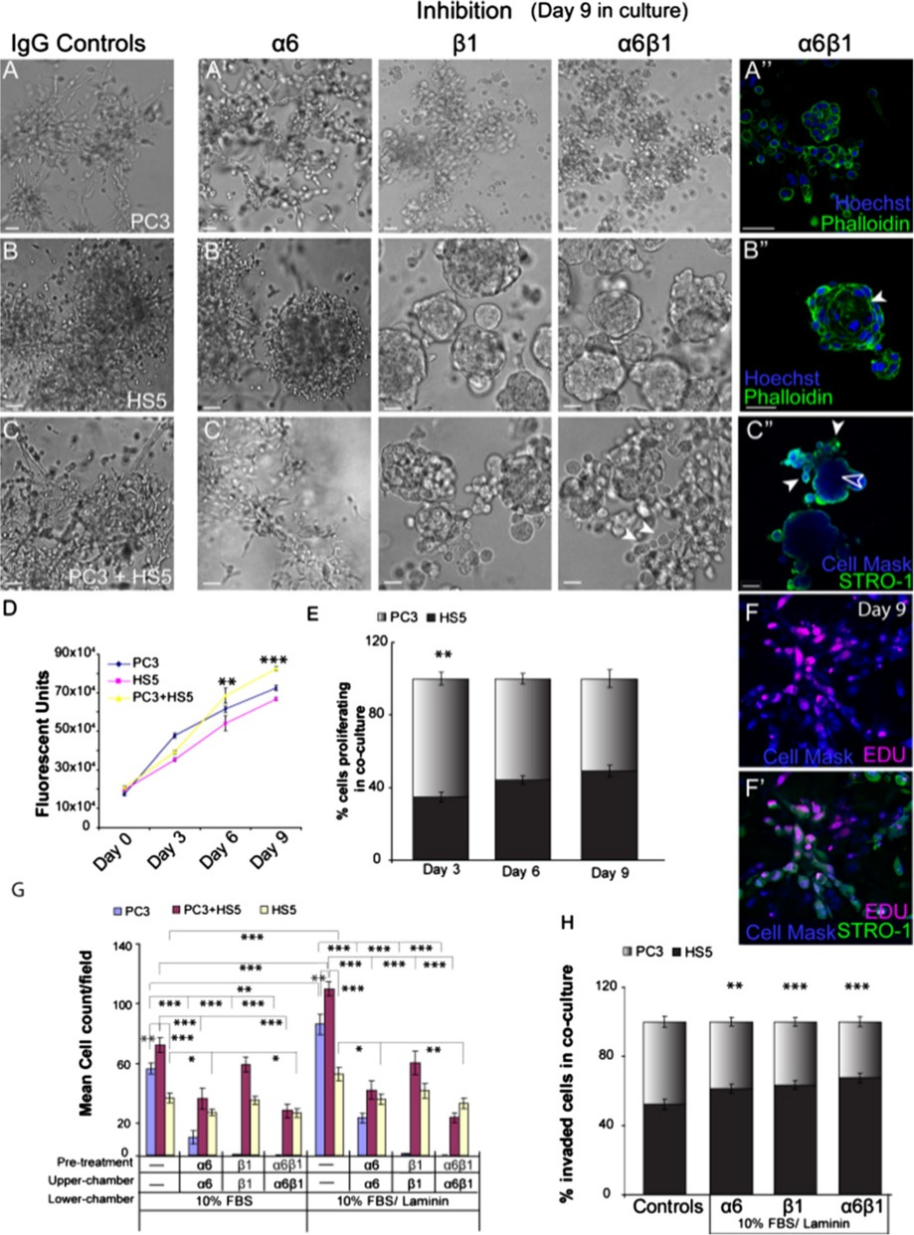
**Integrins mediate morphology and invasive qualities of monocultured and co-cultured cells. (A**-**A”)** PC3 cells grown in the presence or absence of either α6, β1 or both inhibiting antibodies. **(A”)** F-actin staining of PC3 cells in 3D culture. **(B**-**B”)** HS5 cells grown in the presence or absence of either α6, β1 or both inhibiting antibodies. **(B”)** F-actin staining of HS5 cells grown in the presence of both α6 and β1 inhibiting antibodies with acini formation (filled arrowhead). **(C**-**C”)** Co-cultured cells grown in the presence or absence of either α6, β1 or both inhibiting antibodies. **(C”)** In the presence of both α6 and β1 inhibiting antibodies, HS5 cells (STRO-1; green fluorescence) localised to the outer edge (filled arrowhead), while the PC3 cells (Cell Mask blue positive; STRO-1 negative) resided in the centre of the spheroid mass (unfilled arrowhead). **(D)** Quantification of the number of proliferating cells in PC3, HS5 and co-cultured cells over a 9 day period. **(E)** Quantification of the percentage of HS5 and PC3 cells proliferating in co-culture over a 9 day period. **(F**-**F’)** EDU labelling (red fluorescence) of HS5 (STRO-1 positive; green fluorescence) and PC3 cells (CellMask blue positive; STRO-1 negative) in co-culture at day 9. **(G)** Quantification of the number of cells to invade in the presence and absence of integrin inhibitors for PC3, HS5 and co-cultured cells. **(H)**. Quantification of the percentage of invaded HS5 and PC3 cells in co-culture. (**p < 0.01, ***p < 0.001). Error bars denote S.E.M. Scale bars = 40 μm.

When PC3 cells were grown in the presence of the α6 blocking antibody (GoH3), there was little difference in the overall phenotypic appearance of these cells (Figure 2A’) when compared to their IgG controls (Figure 2A). In the presence of β1 (P5B2) or a combination of the α6 and β1 blocking antibodies, PC3 cells displayed a remarkable change in phenotypic structure, losing their stellate morphology, and assuming a more grape-like appearance (Figure 2A’). To investigate further the characteristics of cell junction formation in PC3 cells we carried out immunostaining for F-actin at day 9. PC3 cells treated with α6β1 inhibitors formed rounded grape-like structures with robust cell-cell contacts (Figure 1A”) although no acinar formation or polarisation was evident.

In the presence of α6 blocking antibodies, HS5 cells (Figure 2B’) displayed a similar phenotypic morphology to that of IgG controls (Figure 2B), although at times the boundaries of the spheroid regions were more clearly defined (Figure 2B). In the presence of β1 blocking antibodies, HS5 cells also displayed a remarkable change in phenotypic structure, assuming a well organised and rounded appearance (Figure 2B’). F-actin staining of HS5 cells in these conditions revealed a polarised spheroid structure complete with acinar formation (Figure 2B”; arrowhead).

When cultured together, 3D tumour-stromal cultures displayed disorganised clusters of stellate structures (Figure 2C), with a similar phenotype observed in the presence of α6 blocking antibodies (Figure 2C’). In the presence of β1 or combination α6β1 blocking antibodies, tumour-stromal co-cultures also displayed a reversion of phenotype marked by the presence of rounded polarised masses with additional smaller grape-like structures situated around the periphery (Figure 2C’; arrowheads). F-actin staining of α6β1 inhibited co-cultures revealed that HS5 stromal cells no longer formed acinar as seen in monocultures. Alternatively, they populated the outer regions of the spheroid masses (Figure 2C”; filled arrowheads), while PC3 positive cells populated the inner regions of the spheroid with no acinar formation evident (Figure 2C”; unfilled arrowhead).

These results suggest that β1 integrin can modulate cell-cell contacts and cell-ECM contacts, altering phenotypic morphology in monocultures that are reflective of an epithelial-like reversion. The degree of control exhibited by integrins, however, clearly differs between monocultures and co-cultures as evidenced by the lack of polarisation and acinar formation in HS5 cells in the presence of PC3 cells, suggestive of a more invasive phenotype.

### Proliferation rates in monocultures vs co-cultures

Using an Alamar Blue based proliferation assay conducted over a 9 day period, we were able to determine proliferation rates in 3D for both monocultures and tumour-stromal co-cultures.

Consistent with previous findings [29], in comparison to monocultures of HS5 or PC3 cells, tumour-stromal co-cultures exhibited significantly higher proliferation rates at days 6 and 9 (Figure 2D). To further explore the proliferative behaviour of PC3 and HS5 cells when co-cultured in 3D, an EDU click-it assay was performed to assess the relative contribution of each cell type (Figure 2F-F”). At day 3, in comparison to HS5 cells, PC3 cells proliferated at significantly higher rates, similar to proliferation rates reported for monocultures (Figure 2E). By day 6, both PC3 and HS5 cells were proliferating at similar rates (Figure 2E, F-F”). These results suggest that in the presence of PC3 cells, the proliferative behaviour of HS5 cells is altered when compared to their monoculture counterparts.

### Beta 1 integrin modulates invasive capacity in co-cultures only in the presence of laminin

The ability of cells to metastasise to distal organs is largely mediated by their ability to migrate and invade. Thus we next wanted to ascertain whether there were differences in invasive capacity between monocultures versus tumour-stromal co-cultures and whether α6 and/or β1 integrin may mediate this invasive behaviour. To investigate this we used transwell invasion assays in the presence or absence of α6 and/or β1 function blocking antibodies.

In agreement with previous reports [30], tumour-stromal co-cultures were reproducibly more invasive than monocultures of either HS5 or PC3 cells. These results were observed whether in the presence of FBS or FBS and laminin in the lower chamber wells (Figure 2G). All cultures were observed to invade at significantly higher rates in the presence of laminin (Figure 2G). Inhibition of α6 in PC3 cells significantly decreased their invasive capacity while inhibition of β1 and a combination of α6β1 abolished PC3 cells from invading through the Matrigel and porous membrane (Figure 2G). These results suggest that both α6 and, to a greater degree, β1 integrin subunits positively mediate the invasive capabilities of PC3 cells.

Inhibition of β1 in HS5 cells saw no significant difference in invasive capacity compared with IgG treated controls (Figure 2G). Inhibition of α6 or α6β1 resulted in a significant decrease in invasive capacity, indicating that α6 positively controls invasion in this cell-line. The same results were found when HS5 and PC3 cells were plated together. Inhibition of α6 and a combination of α6β1 to co-cultures saw a consistent decrease in invasive capacity (Figure 2G). However, effects concerning inhibition of β1 on co-cultures were only evident in the presence of its ligand, laminin (Figure 2G).

We next wanted to ascertain the relative contribution of invading stromal and tumour cells in co-culture. To investigate this, transwell invasion assays in the presence or absence of α6 and/or β1 function blocking antibodies with FBS and laminin in the lower chamber wells were used. Following invasion, cells were fixed and each cell type was visualised via staining for STRO-1 and cell mask blue. Unlike their monoculture counterparts, when HS5 cells were in the presence of PC3 cells, their invasive capacity was found to equal that of PC3 cells with 52.3% of invaded cells being HS5 positive (Figure 2H). As expected, inhibition of integrin α6, β1 or combination α6β1 resulted in significantly higher number of HS5 cells invading in comparison to PC3 cells (Figure 2H). In monocultures, PC3 cells were nearly completely abolished but in the presence of HS5 cells, a relatively high percentage of PC3 cells (~ 35%) continued to invade in the presence of β1 or combination α6β1 inhibitors (Figure 2H).

Collectively, these proliferation and invasion results suggest that with the addition of tumour cells, stromal cell behaviour is altered; encouraging increased migratory behaviour and invasiveness. Moreover, in co-cultures, α6 and β1 integrins do not mediate these cellular processes to the same degree as seen in monocultures, indicative that stromal cells may play a protective role against inhibitory elements that may otherwise reduce tumour genesis.

### Alpha 6 and β1 integrins mediate EMT marker expression

Previously it has been shown that inhibiting α6 or β1 integrin activity can induce a re-expression of E-Cadherin in metastatic PCa cell-lines [31]. We then investigated whether α6 or β1 integrin controls the structural homeostasis and expression of important EMT markers including E-Cadherin and N-Cadherin in both monocultures and tumour-stromal co-cultures. Using immunocytochemistry and western blotting techniques, 3D assays were conducted to ascertain EMT expression rates for monocultures including PC3, HS5 and RWPE-1 cells and tumour-stromal co-cultures in the presence or absence of integrin function blocking antibodies.

**Figure 3:**
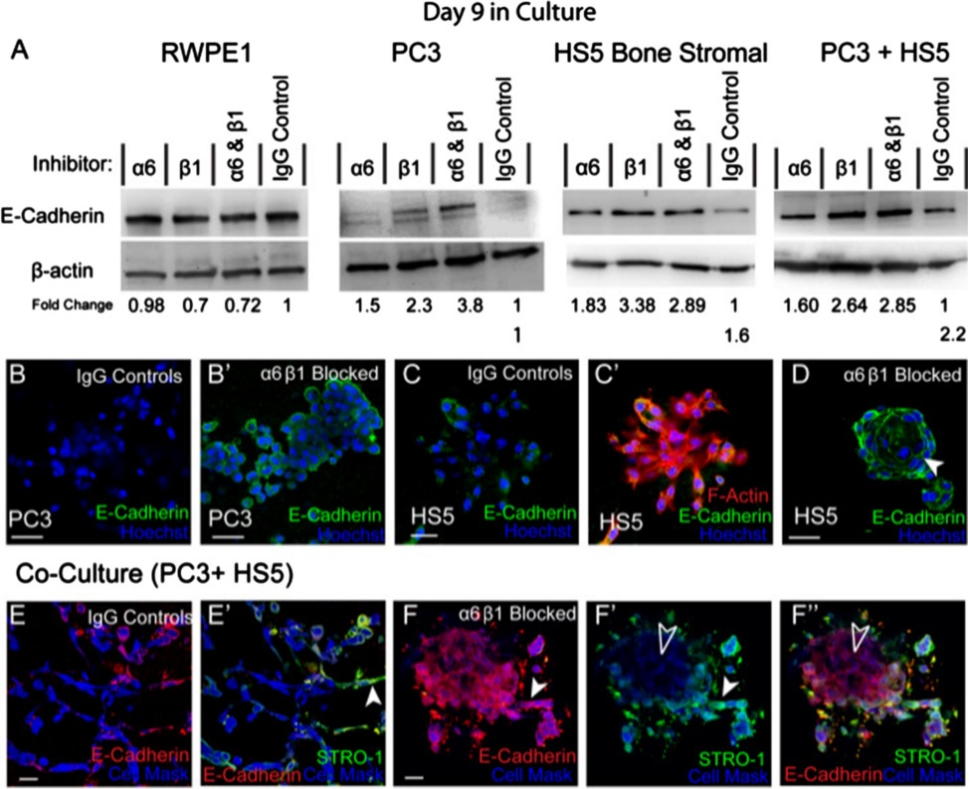
**The expression of E-Cadherin in the presence of integrin inhibitors. (A)** Western blot and densitometric analysis of E-Cadherin expression in RWPE1, PC3, HS5 and co-cultured cells (PC3 + HS5) in the presence and absence of integrin inhibitors. **(B)** Immunostaining of E-Cadherin in PC3 cells. **(B’)** Immunostaining of E-Cadherin in PC3 cells in the presence of α6 and β1 inhibitors. **(C**-**C’)** Immunostaining of E-Cadherin in HS5 cells. **(D)** Expression of E-Cadherin in HS5 cells in the presence of α6 and β1 inhibitors. **(E**-**E’)** Immunostaining of E-Cadherin (red fluorescence) was primarily expressed by HS5 cells (STRO-1; filled arrowhead) in co-culture. **(F**-**F’)** Expression of E-Cadherin in co-cultured cells in the presence of α6 and β1 inhibitors saw an up-regulation on HS5 cells (STRO-1; filled arrowheads) and a re-expression of E-Cadherin on PC3 cells (Cell Mask; unfilled arrowheads). Scale bars = 40 μm.

Western blot analysis revealed that the prostate epithelial cell line, RWPE-1, expressed high protein levels of E-Cadherin that were not altered in the presence of either α6 or β1 integrin blocking antibodies (Figure 3A). In agreement with our previous findings [4], PC3 cells did not express detectable levels of E-Cadherin as confirmed by western (Figure 3A) and immunostaining (Figure 3B). In the presence of α6 blocking antibodies, E-Cadherin expression on PC3 cells was slightly up-regulated, while a 2 fold increase was observed in β1 blocking conditions and a 3 fold increase in combination α6β1 blocking assays (Figure 3A). These results were further confirmed via immunostaining. In the presence of integrin inhibitors E-Cadherin expression was clearly present on the membrane of PC3 cells, indicative of a functional receptor (Figure 3B’).

Similar results were found for HS5 cells. Minimal protein levels of E-Cadherin were found in IgG controls as confirmed by western (Figure 3A) and immunostaining (Figure 3C-C’) results. In the presence of α6 blocking antibodies, E-Cadherin expression on HS5 cells was up-regulated, while a 3 fold increase was observed in β1 blocking conditions and in combination α6β1 blocking assays (Figure 3A). Immunostaining confirmed these results with E-Cadherin clearly present on the membrane of HS5 cells, indicative of a functional receptor (Figure 3D).

In tumour-stromal co-cultures, E-Cadherin expression was up-regulated in IgG controls when compared to monocultures of HS5 or PC3 cells (Figure 3A). Immunostaining revealed that expression was primarily present on HS5 cells (Figure 3E-E’; filled arrowhead). In the presence of α6 blocking antibodies, E-Cadherin protein expression on co-cultured cells was slightly up-regulated, while a 2 fold increase was observed in β1 and combination α6β1 blocking assays (Figure 3A). Immunostaining further confirmed these results with E-Cadherin expression up-regulated on HS5 cells (Figure 3F-F’; filled arrowheads) and re-expressed on PC3 cells (Figure 3F’-F”; unfilled arrowheads).

Collectively, these results confirm that α6, and to a greater degree, the β1 integrin subunit, can mediate E-Cadherin expression and control the structural homeostasis of these cells in both mono and co-culture assays.

**Figure 4:**
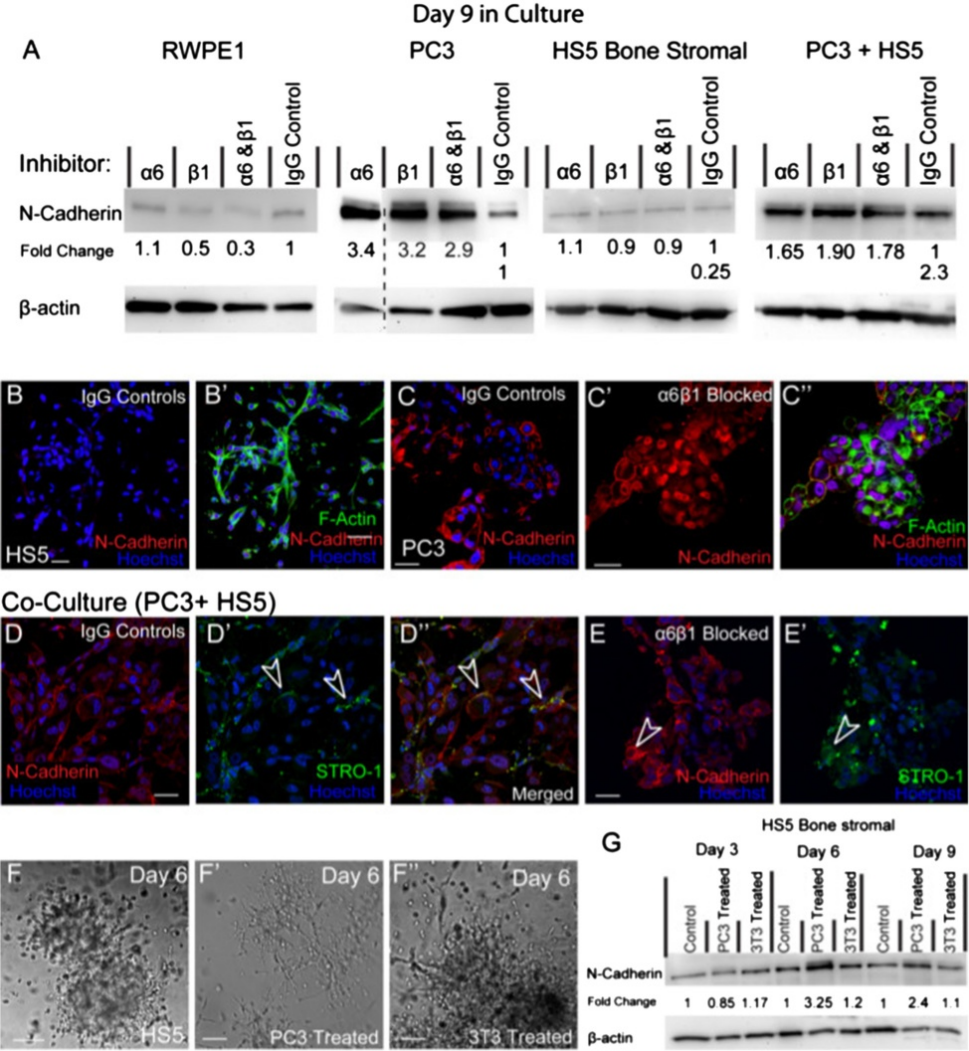
**The expression of N-Cadherin in the presence of integrin inhibitors. (A)** Western blot and densitometric analysis of N-Cadherin expression in RWPE1, PC3, HS5 and co-cultured cells (PC3 + HS5) in the presence and absence of integrin inhibitors. Dashed line indicates samples taken from a separate membrane. **(B**-**B’)** Immunostaining of N-Cadherin in HS5 and **(C)** PC3 cells. **(C’)** Immunostaining of N-Cadherin in PC3 cells in the presence of α6 and β1 inhibitors. **(D**-**D”)** Expression of N-Cadherin in co-cultures, in the presence of PC3 cells, N-Cadherin expression was evident on HS5 cells (STRO-1; unfilled arrowheads). **(E**-**E’)** In the presence of α6 and β1 inhibitors, HS5 cells (STRO-1; unfilled arrowheads) continued to express N-Cadherin. **(F**-**F”)** DIC image of HS5 cells treated with standard media, PC3-treated media or 3T3-treated media. **(G)** Western Blot and densitometric anaylsis of N-Cadherin expression in HS5 cells using standard, PC3-treated and 3T3-treated media over a 9 day period. Scale bar = 40 μm.

RWPE-1 cells exhibited minimal N-Cadherin and in the presence of either β1 or in combination α6β1 blocking assays, N-Cadherin expression was further down-regulated (Figure 4A). HS5 cells expressed minimal levels of N-Cadherin as evidenced by western (Figure 4A) and immunostaining (Figure 4B-B’) with no alterations observed in the presence of integrin function blocking antibodies (Figure 4A). Alternatively, PC3 cells expressed detectable levels of N-Cadherin and in the presence of α6, β1 or a combination of both integrin inhibitors, expression was up-regulated 3 fold (Figure 4A). Immunostaining revealed a redistribution of N-Cadherin expression on PC3 cells from primarily membrane bound on IgG controls (Figure 4C) to cytoplasmic and nucleic on cells treated with α6, β1 or α6β1 inhibitors (Figure 4C’-C”), indicative of a non-functional receptor. These results suggest that both α6 and β1 integrin subunits are vital to the functional presentation of N-Cadherin to the membrane in PC3 cells.

In co-cultures, N-Cadherin expression was present as observed by both western (Figure 4A) and immunostaining (Figure 4D-D”). It became evident that once plated with PC3 cells, HS5 cells re-expressed N-Cadherin that was clearly present on the membrane (Figure 4D’-D”; arrowheads). Co-cultures treated with α6, β1 or a combination of α6β1 inhibitors resulted in an up-regulation of N-Cadherin expression (Figure 4A). In these conditions, HS5 cells continued to re-express membranous N-Cadherin (Figure 4E-E’; arrowheads). Moreover, unlike their mono-cultured counterparts, PC3 cells in co-culture were found to express membranous N-Cadherin, suggesting that in the presence of HS5 cells, integrin inhibition no longer rendered N-Cadherin non-functional. These results suggest that HS5s may provide a “protective” mechanism that encourages the retention of functional mesenchymal properties known to encourage tumour progression.

We next wanted to ascertain whether the up-regulation of N-Cadherin expression in HS5 cells was due to soluble factors excreted by PC3 cells in co-culture assays. To investigate this HS5 cells were treated with PC3 treated media over a 9 day time-course. In comparison to untreated HS5 cells (Figure 4F), HS5 cells grown in PC3 treated media lost their organised phenotype by day 6 in culture and formed irregular shaped clusters with stellate radiating tubular processes, consistent with a metastatic cell-line (Figure 4F’). These results were PC3 specific as HS5 cells grown in embryonic fibroblastic (3T3) treated media (Figure 4F”) were unaffected. Moreover, western results confirmed an up-regulation of N-Cadherin expression in HS5 cells when treated with PC3 treated media with a 3 and 2.4 fold increase at days 6 and 9, respectively (Figure 4G).

### Beta 1 integrin mediates vimentin expression in 3D monocultures

**Figure 5:**
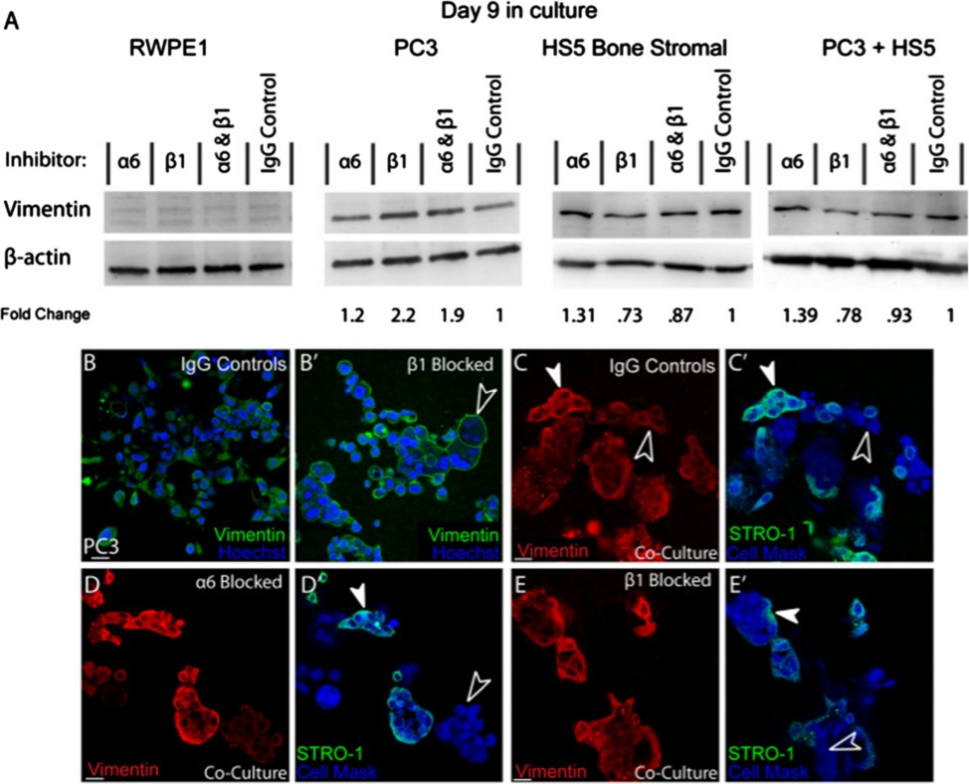
**The expression of vimentin in the presence of integrin inhibitors. (A)** Western blot and densitometric analysis of vimentin expression in RWPE1, PC3, HS5 and co-cultured cells (PC3 + HS5) in the presence and absence of integrin inhibitors. **(B**-**B’)** Immunostaining of vimentin in PC3 cells under **(B)** control and **(B’)** β1 inhibitor conditions where vimentin was re-distributed to the membrane (unfilled arrowhead). **(C**-**C’)** Expression of vimentin in co-cultures. Vimentin was present in the cytoplasm of HS5 (STRO-1; filled arrowheads) and PC3 (Cell Mask; unfilled arrowheads) cells. **(D**-**E’)** In the presence of α6 or β1 inhibitors, HS5 (STRO-1; filled arrowheads) and PC3 (Cell Mask; unfilled arrowheads) cells continued to express vimentin in the cytoplasm. Scale bars = 40 μm.

Consistent with an epithelial phenotype, RWPE1 cells did not express detectable levels of vimentin (Figure 5A). Alternatively, invasive (PC3) and mesenchymal (HS5) cell types expressed vimentin with similar levels recorded in co-culture assays (Figure 5A). In the presence of α6 blocking antibodies, expression of vimentin was not altered on PC3, HS5 or co-cultured cells. Alternatively, in the presence of β1 blocking antibodies, vimentin was up-regulated 2 fold in PC3 cells, while there was minimal effect on total protein expression found in monocultured HS5 cells (0.73) or in co-cultures (0.78; Figure 5A). Similar results were found in cells grown in the presence of α6β1 inhibitors (Figure 5A).

Immunostaining of monocultured PC3 cells revealed that in IgG controls, vimentin expression was evident within the cytoplasm and cytosol of the cell (Figure 5B), indicative of a functional intermediate filament (IF) protein. Alternatively, when treated with β1 or combination α6β1 inhibitors, vimentin expression was redistributed to the membrane of PC3 cells (Figure 5B’). These results suggest that β1 integrin, in this specific cell line, is involved in maintaining the functional localisation of this receptor to the cytosol of the cell.

In HS5 cells, vimentin distribution remained within the cytoplasm and cytosol of the cell and this distribution remained unaltered in the presence of any integrin inhibition parameters (results not shown). Similarly, when co-cultured, HS5 and PC3 cells retained a distribution pattern consistent with a functional IF receptor (Figure 5C-C’; filled arrowhead (HS5), unfilled arrowheads (PC3)). Moreover, in co-cultures, PC3 cells were found to express functional cytosolic vimentin in the presence of β1 or combination α6β1 inhibitors (Figure 5D-E’; filled arrowhead (HS5), unfilled arrowheads (PC3)). These results provide further evidence that HS5s in this model help to retain mesenchymal properties known to encourage tumourgenesis.

### Alpha 6 and β1 integrins mediate chemokine CXCR7 receptor expression in tumour-stromal co-cultures

Previously, we have found that CXCR4 chemokine receptors are highly expressed on the stellate processes exhibited by PC3 cells in 3D culture [4]. Following on from these results, we next wanted to ascertain the expression rates of another important chemokine receptor CXCR7 and whether α6 and/or β1 integrins mediate the expression of these receptors.

**Figure 6:**
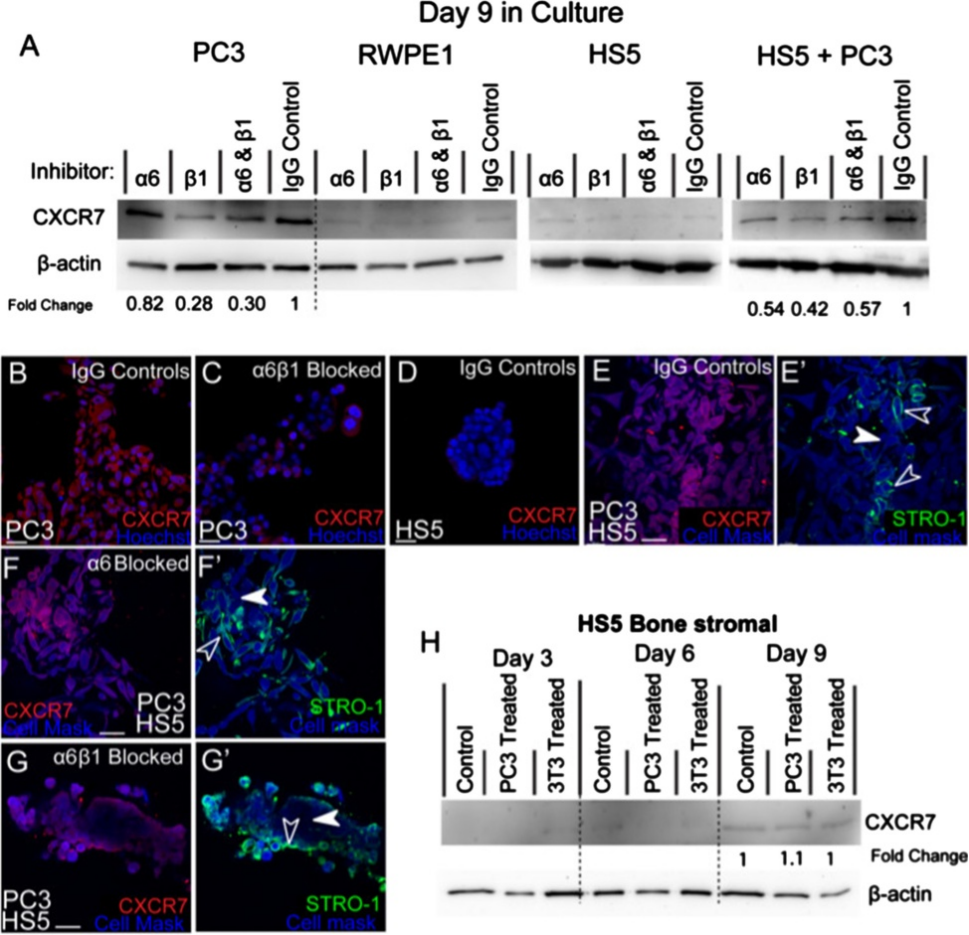
**The expression of CXCR7 in the presence of α6 and β1 integrin inhibitors. (A)** Western blot and densitometric analysis of CXCR7 expression in RWPE1, PC3, HS5 and co-cultured cells (PC3 + HS5). **(B)** Immunostaining of CXCR7 expression in PC3 cells in the presence of IgG control antibodies and **(C)** α6 and β1 inhibitors. **(D)** Immunostaining of CXCR7 expression in HS5 cells. **(E**-**E’)** Immunostaining of CXCR7 in co-cultures where HS5 cells (STRO-1) were found to re-express CXCR7 (unfilled arrowheads) at similar levels to that found on PC3 cells (filled arrowhead). **(F**-**F’)** In co-cultures, HS5 (STRO-1; unfilled arrowheads) and PC3 cells (Cell Mask; filled arrowheads) continued to express CXCR7 in the presence of α6 inhibitors. **(G**-**G’)** In the presence of α6 and β1 inhibitors, HS5 cells (STRO-1; unfilled arrowheads) continued to express CXCR7 at higher levels than found on PC3 cells (filled arrowhead). **(H)** Western blot and densitometric analysis of CXCR7 expression in HS5 cells using standard, PC3-treated, and 3T3-treated media over a 9 day period. Scale bars = 40 μm.

In 3D, PC3 cells consistently expressed CXCR7 as evidenced by western (Figure 6A) and immunostaining (Figure 6B). In comparison to IgG controls, (Figure 6A; B), down regulation of CXCR7 expression was evident in the presence of β1 or a combination of α6β1integrin inhibitors, while inhibition of α6 saw no change (Figure 6A; C). These results suggest that on monocultured PC3 cells, CXCR7 expression is positively mediated by β1 integrin.

Prostate epithelial cell-line RWPE-1 did not express detectable levels of CXCR7 (Figure 6A), nor did monocultured HS5 cells (Figure 6A, D). However, when co-cultured, HS5 cells were found to re-express CXCR7 (Figure 6E-E’; empty arrowheads) at levels similar to that found on PC3 cells (Figure 6E-E’; filled arrowhead). Westerns revealed that in the presence of α6, β1or a combination of inhibitor antibodies, CXCR7 expression was consistently down-regulated (Figure 6A). Dissimilar to monocultured PC3 cells, in co-cultures, α6 was now found to positively mediate CXCR7 expression.

Immunostaining revealed that in α6 inhibited co-cultures both PC3 (Figure 6F-F’; filled arrowheads) and HS5 cells (Figure 6F-F’; unfilled arrowheads) continued to express CXCR7 at similar levels, however in β1 and α6β1 inhibitor assays, CXCR7 was predominately expressed by HS5 cells (Figure 6G-G’; unfilled arrowheads), with little expression noted on PC3 cells (Figure 6G-G’; filled arrowheads). These results suggest that similar to monoculture conditions, β1 integrin continues to mediate CXCR7 expression on PC3 cells in co-culture.

To verify whether soluble or contact mediated factors associated with PC3 cells could regulate the re-expression of CXCR7 on HS5 cells, HS5 cells were grown over a 9 day time-course in the presence or absence of PC3 treated media. When HS5 cells were challenged with PC3 or 3T3 treated media, no evident alteration in CXCR7 expression was found (Figure 6H). Furthermore, CXCR7 expression was barely detectable by day 9 in culture. These results suggest that soluble factors excreted by PC3 cells do not mediate up-regulation of CXCR7. It is likely that other factors including endocrine cell-cell and cell-ECM contact mediation may regulate endogenous up-regulation in co-cultured HS5 cells.

## Discussion

In agreement with previous findings [6,29], our results suggest that addition of stromal cells to metastatic PCa cells in 3D culture can accelerate cancer growth and invasion. Through soluble and contact mediated mechanisms, PC3 and HS5 cells reciprocally interact to facilitate tumour growth by up-regulating EMT markers and chemokine receptors known to mediate bone metastatic dissemination. In addition, we demonstrate for the first time that both α6 and β1 integrins mediate invasion, EMT protein expression and phenotypic homeostasis in these co-cultures.

### Morphological changes in HS5 cells in co-culture

Utilising both DIC and fluorescence microscopy we and others [3,4] have confirmed that when grown in Matrigel the PCa cell-line, PC3 formed structures consistent with an invasive phenotype while HS5 cells formed structures consistent with a non-malignant phenotype . When cultured together, the phenotypic characteristics of monocultured HS5 cells are altered becoming a highly disorganised arrangement of cells characterised by long chains of stellate processes, consistent with a highly invasive phenotype. In co-cultures, both cell types formed cell-cell contacts. These results coincide with others who have shown that cancer-stromal interactions can lead to spontaneous fusion between the two cell types [32].

When co-cultured with another metastatic cell line, DU145, HS5 cells were not seen to alter in phenotype with both cell types forming isolated cell specific masses. Similar results have been shown where bone derived metastatic cancer cells adhere more avidly to bone-marrow derived endothelial cells in comparison to endothelial cells harvested from non-target organs [33]. Our results are consistent with the idea that tumour-stromal micro-environments are highly niche specific. Both PC3 and HS5 cells are derived from the bone micro-environment where similar ECM molecules and gene expression programs are established. Alternatively, DU145 cells are derived from the brain in the central nervous system where ECM parameters are very different [34].

### Inhibition of β1 integrin results in phenotypic reversion

To the best of our knowledge, this is the first time that the effect of α6 and β1 integrin function-blocking antibodies has been tested against tumour-stromal co-cultures in 3D. Here we have shown that in the presence of antibody inhibitors for β1 integrin, PC3, HS5 and tumour-stromal cell co-cultures all displayed alterations in their phenotypic appearance. Both PC3 and tumour-stromal co-cultures displayed a partial reversion with no acinar formation present, while HS5 cells cultured alone displayed a drastic reversion to a complete epithelial type, marked with prominent acinar formation. Similar results have been reported for a highly metastatic PCa cell-line M12; acinar formation was evident after inhibition of either β1 or α6 integrin subunits [31]. In contrast, we found that inhibition of α6 did not clearly mediate obvious phenotypic changes in these cell-lines and in part could be explained by the promiscuous nature of the β1 subunit. It is known that the β1 subunit has over 8 known alpha subunit partners with both α2β1 and α5β1 actively implicated in the tumour-bone stromal processes [12,13]. Therefore in our β1 inhibitor assays, it is assumed that we are in part preventing the activation of all these alpha subunits. Alternatively when we inhibit the α6 subunit, it is highly likely that the partnering of the β1 subunit to other known alpha subunits is altered and possibly encourages activation of both α2 and α5 subunits. As such, understanding how the inhibition and/or activation of one subunit can affect the coupling of other known heterodimer partners in tumour development will be imperative in establishing therapeutics targets and is the work of future studies.

Noteworthy is the inability of β1 inhibition to completely revert the phenotype of HS5 cells once co-cultured with PC3 cells. These results are consistent with the idea of rapid phenotypic plasticity where human bone stromal cells undergo permanent cytogenetic and gene expression changes, altering their cell-ECM profiles in the presence of metastatic cells [5].

### Co-cultures display altered invasion and proliferation rates

Consistent with previous findings [29,30] co-cultured cells proliferated and invaded at significantly higher rates in comparison to PC3 or HS5 cells plated in isolation. Of interest was the apparent up-regulation of proliferation rates following 3 days in culture for mono and co-cultured cells. This time-course correlates with progressive changes in cell-cell interactions and various genes involved in lipid/steroid metabolism, adhesion, ECM turnover and development/differentiation known to occur in a 3D *in vitro* micro-environment [3]. It is therefore likely that the changes noted in proliferative rates were a result of interactions and cross-talk between growth factors and hormones being released within the enhanced paracrine network of the co-culture structure. This would explain in part studies [35] that have reported no apparent differences in proliferation rates in co-cultures of PC3 and HS5 cells using 2D monolayer assays where appropriate cell-cell and cell-ECM contacts are not formed.

Supported by recent *in vitro* studies [31,36] and knockout strategies in transgenic mouse tumour models [37], we have shown that integrins can control tumour growth. Collectively, our results demonstrate that both α6 and β1 integrins mediate invasion of monocultured and tumour-stromal co-cultured cells in 3D. Similar to previous results regarding integrins α2β1 and α5β1 [12,13], this is the first report to establish that α6β1 can actively facilitate tumour-bone stromal interactions *in vitro*. In monocultures, α6 is highly influential in positively mediating invasion in HS5 cells, while both β1 and α6 integrins positively mediate these functions in PC3 cells. When these cell lines are cultured together however, the degree to which integrins mediate invasion are attenuated, favouring a more tumourgenic niche. These results could in part explain why certain integrin therapeutics have been less than convincing in clinical trials [38]. Similar to findings utilising perineural invasion models in PCa [39], we have also shown that co-cultures display an increased dependency on the ECM component, laminin, during invasion. Establishing a comprehensive understanding of the increased laminin dependent invasion of both tumour and stromal cells may provide a further framework for developing pertinent biomarkers or targeted therapies.

### Alterations in endogenous expression of EMT markers and chemokine receptors in co-culture

**Figure 7:**
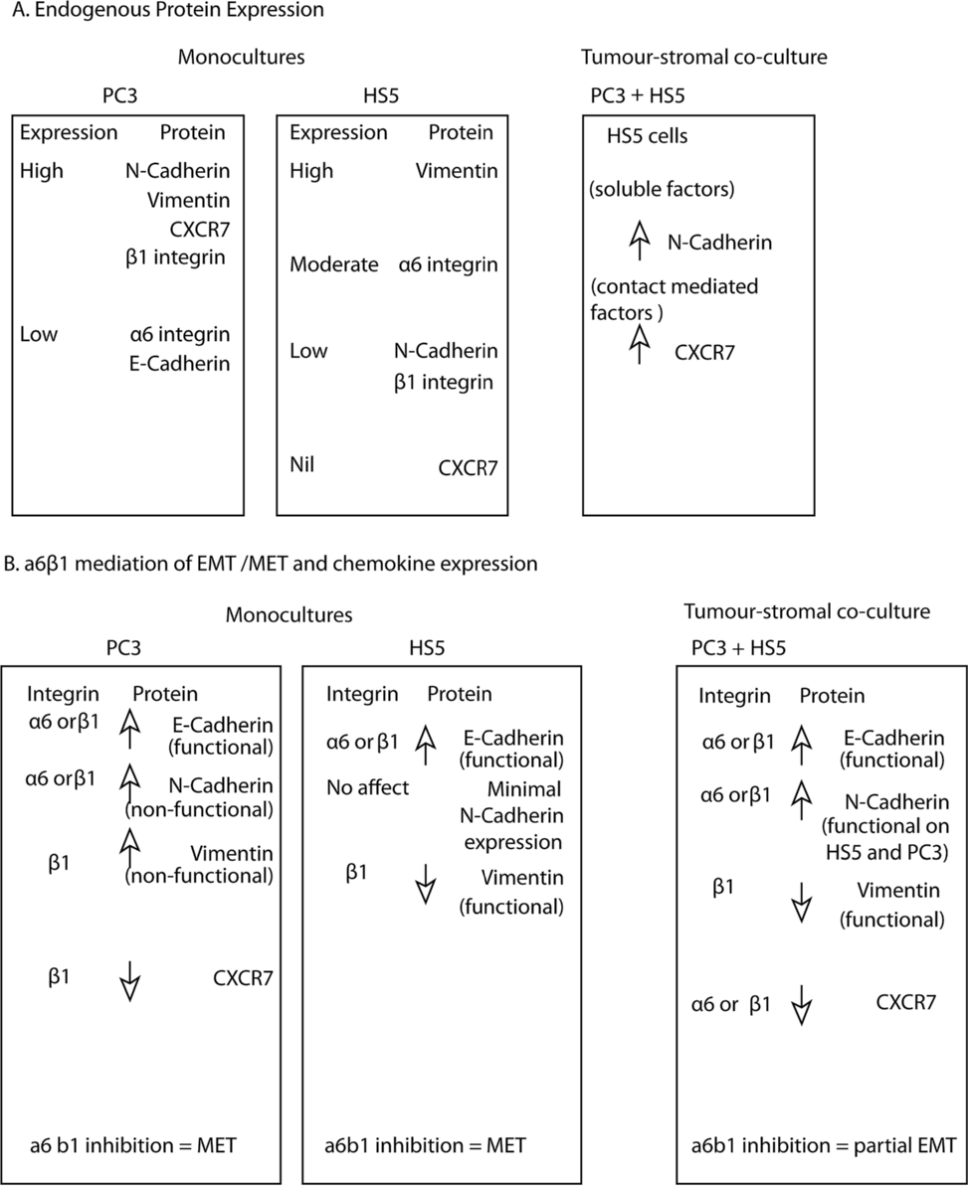
**Summary of endogenous and integrin mediated expression profiles of monocultured and co-cultured cells. (A)** Summary of endogenous protein expression in PC3, HS5 and co-culture models. **(B)** Summary of integrin mediated protein expression in monocultures of PC3, HS5 and co-culture models.

Our results regarding endogenous expression of EMT markers and chemokine receptors in monocultured and co-cultured cells is summarised in Figure 7A. In monocultures, PC3 cells exhibit profiles consistent with a highly metastatic phenotype, while HS5 cells expressed profiles consistent with a non-cancerous mesenchymal phenotype. When co-cultured with PC3 cells, HS5 cells re-expressed N-Cadherin and CXCR7, proteins that are known to accelerate cancer growth at the primary tumour site and support metastatic colonisation in distal organs [40,41].

One of the hallmarks of EMT is the loss of the E-Cadherin, and the concomitant increase in expression of the mesenchymal cell-cell adhesion molecule N-Cadherin, a process also known as the Cadherin switch [42,43], which can provoke cell migration and invasion in breast cancer cells [44,45]. Here we report for the first time that soluble factors excreted by PC3 cells can alone mediate the re-expression of N-Cadherin in HS5 cells. Functionally, this up-regulation is known to cause a change in the adhesive properties of cells and in the case of tumour cells, lose their affinity for their epithelial neighbours, a mechanism that encourages metastatic seeding and colonisation [43].

Further studies are now needed to verify the identity of these soluble molecules responsible for this up-regulation in N-Cadherin and the direct functional consequences of these alterations. A large number of growth factors and their activated signal transduction pathways are known to provoke the Cadherin switch including transforming growth factor β (TGFβ; [46]), hepatocyte growth factor (HGF; [47]), insulin-like growth factor (IGF; [48]), fibroblast growth factor (FGF; [49]) and Notch signalling [50].

In addition to soluble factors, there are a plethora of contact mediated variables that could account for the re-expression of CXCR7 in co-cultured HS5 cells. One possibility is the modulation of chemokine receptors through hypoxic conditions, which is known to induce cancer cell expression of c-Met, the bona fide receptor of HGF, and CXCR4, the signalling receptor of the chemokine CXCL12 (SDF1), and further stimulate cancer cell migration and dissemination [51,52].

### Alpha 6 and β1 integrins mediate EMT proteins and CXCR7 expression in co-cultures

We report here that both α6 and β1 integrin subunits can influence expression rates of important EMT markers (E-Cadherin, N-Cadherin and vimentin) and chemokine receptor CXCR7 in both monocultured and co-culture assays. Our results regarding integrin mediated changes in these proteins is summarised in Figure 7B. Taken together, our results suggest that inhibition of α6 and β1 integrins can mediate a MET program in monocultured cells (See Figure 7B), while integrin mediation in co-cultures is clearly altered with the re-establishment of functional N-Cadherin and vimentin expression on PC3 cells, consistent with an EMT program. These results suggest that HS5 cells may play a role in maintaining functional homeostasis of N-Cadherin and vimentin expression on PC3 cells and as such maintain a higher incidence of mesenchymal attributes. These results highlight the importance of stromal cells in contributing to the effectiveness of integrin centred therapeutics.

Of interest was the clear redistribution of N-Cadherin and vimentin in monocultured PC3 cells when treated with β1 inhibitors. The distribution patterns of these markers were indicative of a reduced junctional and IF protein, respectively. However, the degree to which E-Cadherin in these cells may then activate the Cadherin-catenin complex to mediate the metastatic phenotype needs further clarification. Previous studies have shown that with a decrease in junctional E-Cadherin protein, catenins become localized to the nucleus where they activate the transcription of proto-oncogenes, stimulating mitosis [53].

## Conclusions

Using 3D tumour-stromal co-cultures we have shown that the addition of bone derived stromal cells to metastatic PCa cells helps support tumour growth and protects PC3 cells from integrin mediated alterations associated with MET. Reciprocally, we have also found that the addition of PC3 cells results in significant up-regulation of invasive and proliferative behaviour in addition to re-expression of N-Cadherin and CXCR7 on HS5 cells. Further studies now need to evaluate the cross talk that occurs between these two compartments on a systematic, cellular and molecular basis and will likely lead to identification of new targets for therapy.

## Materials and methods

### PCa Cell Lines

Cell lines were purchased from ATCC and were passaged for less than 4 weeks during any given assay performed for this paper. ATCC routinely use COI for interspecies identification and STR analysis (DNA profiling) for intra-species identification for all cell-lines. The PCa cell lines (DU145, PC3), Bone Stromal Cell-line (HS5) and the 3T3 fibroblast cell-line were maintained in RPMI-1640 (Sigma-Aldrich), supplemented with 10% fetal bovine serum (FBS, Gibco) and the prostate epithelial cell-line RWPE-1 was maintained in Keratinocyte Serum Free Media (KSFM, Gibco) supplemented with 20 mg/mL bovine pituitary extract (BPE) and 0.2 ng/mL epidermal growth factor (EGF). All cells were propagated in standard cell culture conditions (5% CO_2_, 37°C) in cell cultured treated T75 Flasks (Flalcon). Media was replenished every 3 days. Once cells had reached 80-90% confluency, cells were replated (1/10) in T75 flasks. After 10-12 passages, cells were discarded.

### 3D cultures and tumour-stromal co-cultures

For miniaturised 3D cultures, 45 μl phenol red free (PRF) Matrigel™/culture medium (70%: BD Biosciences) was added to 96 well plates and polymerised at 37°C with 5% CO_2_ for 1 hr. Cultures of cell-lines including RWPE-1, PC3, DU145 and HS5 cells were seeded at ~5000 cells per well and co-cultures containing both PC3 and HS5 cells were plated together at ~2500 cells each (1:1 ratio) per well and maintained in standard culture conditions. Media was carefully removed and replenished every 3 days. Cultures were maintained for up to 9 days.

### 3D bulk cultures for protein extraction

Protein extraction for western blotting was obtained from 3D Matrigel cultures grown in 12-well plates. For 3D cultures, 450 μl PRF Matrigel™/culture medium (70%: BD Biosciences) was added per well and allowed to polymerise at 37°C with 5% CO_2_ for 1 hr. Single cell cultures were then seeded at ~10000 cells per well while co-cultures containing HS5 and PC3 cells were plated at ~5000 cells each (1:1 ratio) per well and media was replenished every three days. After 3, 6 and 9 days in culture, 3D bulk cultures were extracted using Cell Recovery Solution (CRS: BD Biosciences) as per the manufacturer’s instructions. Cell pellets were then lysed and western blotting techniques were carried out.

### Integrin α6 and β1 inhibition assays

In order to block α6 or β1 integrin subunits, well established functional blocking antibodies were diluted (1.5 μg/mL) directly into the 3D matrix as follows: 1. α6: GoH3 (R&D Systems), 2. β1: P5B2 (R&D systems), 3. α6 and β1 and 4. IgG isotope controls (R&D Systems). Cells were then seeded and grown for 9 days in culture in miniaturised 96 well or a bulk 12 well plate format. Functional blocking antibodies and IgG isotope controls were replaced during media changes every 3 days at 1.5 μg/mL concentration. Miniaturised 3D cell cultures were then washed and fixed with 4% paraformaldehyde (PFA) and immunocytochemistry was undertaken. For inhibition assays carried out in a 12 well format, cells were extracted using CRS and western blotting techniques were undertaken.

### Western blotting

Protein was collected from cells at days 3, 6 and 9 from bulk 3D cultures. Treated cells were lysed in ice-cold RIPA buffer (75 mM TrisCl pH 8, 150 mM NaCl, 0.1% SDS, 1% Triton-X-100, 0.5% deoxycholic acid) containing protease inhibitors (Roche), incubated at 4°C for 30 mins prior to centrifugation at 14,100 g for 20 mins to pellet cell debris. The supernatants were then assayed for protein concentration using DC Protein Assay (Bio-Rad), and equal amounts of protein were loaded onto SDS-PAGE gels for electrophoresis. The protein was then transferred to Polyvinylidene Fluoride (PVDF) membranes in transfer buffer (25 mM Tris Base, 200 mM glycine containing 15% methanol) for 30 mins using a Bio-Rad Turbo-Blot system. PVDF membranes were blocked using 5% non-fat milk powder for 1 hr, washed with TBST and primary antibodies were applied in blocking buffer as follows: mouse anti-E-Cadherin (2 μg/mL, Invitrogen), anti-human integrin α6/CD49f (1 μg/mL; R&D Systems), anti-human integrin β1/CD29 (0.2 μg/mL; R&D Systems), goat anti-human vimentin (0.2 μg/mL, R&D Systems), rabbit anti-CXCR7 (0.2 μg/mL; Abcam) and anti-human N-Cadherin (0.5 μg/mL, R&D Systems) applied overnight (O/N) at 4°C. Membranes were then washed and HRP-conjugated secondary antibodies (Bio-Rad) applied for 1 hr at 4°C prior to washing and imaging on a Versa Doc (Bio-Rad) imaging station. Membranes were stripped and re-probed for β-actin in the case of vimentin, CXCR7 and CXCR4, whereas E-Cadherin, N-Cadherin and integrin α6, β1 membranes were directly probed for β-actin. Densitometric analysis was performed using Image Lab software and expressed as a fold change in relation to loading controls and normalised against β-actin.

### Immunocytochemistry

Miniaturised 3D cultures of PCa cells (DU145, PC3, RWPE-1, HS5) and co-cultures (HS5 + PC3) grown in 384 well format were washed (3 × 5 mins PBS) and fixed with 4% PFA for 20 mins. For immunofluorescence labelling, cells were washed (3 × 5 mins PBS), permeabilised and blocked O/N with 2% BSA, 0.1% Triton-X, 0.05% TWEEN20 at 4°C. Cells were further washed (1 × 5mins PBS/0.1%TX, 2 × 5 mins PBS) and the following primary antibodies were applied O/N at 4°C in blocking buffer: mouse anti-E-Cadherin (5 μg/mL, Invitrogen), anti-human Integrin β1/CD29, Anti-human Integrin α6/GoH3, Goat anti-human vimentin, anti-human N-Cadherin, mouse anti-STRO-1 (5 μg/mL, R&D Systems) and mouse anti-CXCR7 9C4 (5 μg/mL, MBL International). Cells were washed with PBS (3 × 5 mins), incubated with appropriate secondary antibodies (5 μg/mL 488 goat anti-mouse, 5 μg/mL 594 goat anti-rat, 5 μg/mL 594 goat anti-rabbit), nuclear stain Hoechst (1/1000, Invitrogen), filamentous actin stains: Texas Red Phalloidin or 488 Phalloidin (1/80, Invitrogen) and Cell Mask Blue (1/500, Invitrogen) for 4 hrs at room temperature (R/T). Cells were washed then imaged using PerkinElmer Opera™ Confocal Imager and an Olympus IX-81 Scanning Confocal microscope.

### Proliferation assays of mono and co-cultured 3D cells

To assess cell proliferation in mono and co-cultured 3D cells, assays were performed in 384-well plates using Alamar Blue reagent (Invitrogen). TC-treated Falcon 384-well plates (BD Biosciences) were applied with 15 μl of 70% Matrigel (diluted in cold SFM) and left to polymerise for 2 hrs at 37°C, 5% C0_2_ and 95% humidity. Mono culture (HS5 or PC3 cells) were plated at ~800 cells/well and co-cultures (HS5 + PC3 cells) were plated at ~400 cells/well each to make a total of ~800 cells/well in 50 μL complete medium per well and left to adhere O/N at 37°C, 5% CO_2_ and 95% humidity. A baseline reading was taken 24 hours after plating (Day 0), and readings were obtained on assay days 3, 6 and 9 through application of 5 μl Alamar Blue per well, achieving a final concentration of 10% (v/v). After addition of Alamar Blue cells were further incubated for 4 hrs at 37°C, 5% CO_2_ and 95% humidity, before plates were read on the Envision Plate Reader (Perkin Elmer) using fluorescence excitation/emission settings of 530 nm/ 595 nm. To investigate the relative contribution of proliferating HS5 and PC3 cells in co-culture, cells were treated with Click-iT EdU HCS 594 kit (Invitrogen) at days 3, 6 and 9 in culture. After incubation with the EdU compound in serum free media (10 μM, 24 hr), cells were fixed with PFA, washed (3×5 mins PBS) and a 594 fluorescent azide solution (100 mM TrisCl pH 8.5, 0.75 mM CuSO_4_, 50 mM Fluorescent Azide, 75 mM Ascorbic Acid, Milli-Q water) was applied O/N at 4°C in blocking buffer along with STRO-1 (5 μg/mL, R&D Systems) antibody. The following day a general cytoplasmic and nuclear stain (Cell Mask Blue 1/1000 and 1.5 hrs, Invitrogen) and a secondary antibody (5 μg/mL 488 goat anti-mouse) was applied for 4 hrs at R/T. Cells were finally washed (3×5 min PBS) and imaged using an Olympus confocal and results were analysed using Imaris volume and spots.

### Transwell cell invasion assays

To investigate the role integrin α6 and β1 play in mediating invasive cell behaviour, transwell cell invasion assays (Corning, polycarbonate, 8.0 μm pore size) were employed. Two days prior to each invasion assay, PC3 and HS5 cells were seeded in 6 well plates at a density of 500,000 cells/well and co-culture (PC3 + HS5) cells were seeded together at a 1:1 ratio to a total 500, 000 cells/well and left to adhere O/N at 37°C, 5% C0_2_ and 95% humidity. The following day, cultures were serum starved for 16-24 hours in the presence of integrin function blocking antibodies: 1.5 μg/mL of α6; GoH3, 1.5 μg/mL of β1; P5B2, α6 and β1 and 1.5 μg/mL of mouse IgG isotope controls. On the day of the assay, cells were harvested with accutase and seeded at a density of ~150,000 cells per transwell insert in a volume of 200 μl SFM with the addition of integin inhibitors: 1. 1.5 μg/mL of α6; GoH3, 2. 1.5 μg/mL of β1; P5B2, 3. α6 and β1 or 4. 1.5 μg/mL of mouse IgG isotope controls. Prior to seeding cells, 20 μl of GFR Matrigel (1:5 dilution/SFM) was applied to the transwell insert and polymerised for 1 hr at 37°C, 5% CO_2_ and 95% humidity. The undersides of the transwell inserts were then coated with 4 μg of laminin (Invitrogen) to encourage attachment of migrated cells. For coating, a 1 mg/mL laminin stock solution was diluted 1/12.5 in warmed PBS, and 50 μl of this solution was dispensed onto each insert and left to evaporate at RT. The inserts were then washed in PBS and equilibrated in SFM for 1 hr at 37°C, 5% CO_2_ and 95% humidity before cells were seeded onto the prepared transwell inserts. Following addition of cells, 600 μl SFM was added to the lower chamber with or without 10% FBS or 10% FBS + 30 μg/mL of laminin and the plates were incubated at 37°C, 5% CO_2_ and 95% humidity for 32 hrs to allow for cell invasion to occur.

Cell invasion was then quantified through staining with crystal violet. Invaded cells were fixed with 100% Methanol for 10 mins at -20°C, prior to application of crystal violet staining mixture (0.5% crystal violet in 20% Methanol) for 30 mins to allow visualisation of cells. The non-invaded cells on the upper surface of the insert were removed with a cotton swab, the inserts washed in purified water and left to air dry. Cell invasion was quantified using images obtained on the InCell 1000 (GE) and processed by an automated script generated by InCell Developer. Counts were averaged between 3 assay replicates.

To further quantify the relative proportion of invading HS5 and PC3 cells in co-culture, experiments were repeated as outlined above and cell invasion was quantified through staining with primary antibody STRO-1 (5 μg/mL, R&D Systems) for 2 hrs at R/T followed by a general cytoplasmic and nuclear stain (Cell Mask Blue 1/1000 and 1.5 hrs, Invitrogen) and a secondary antibody (5 μg/mL 488 goat anti-mouse) application for 2 hrs at R/T. Cells were finally washed (3×5 min PBS), membrane inserts carefully removed from the transwells, placed on a glass slide and imaged using an Olympus confocal and results were analysed using Imaris volume and spots.

### HS5 cultures treated with PC3 and 3T3 conditioned media

For these assays, PC3 and 3T3 fibroblast cell-lines were propagated in T75 flasks for a minimum of 48 hrs in RPMI complete media and maintained at 37°C in standard cell culture conditions (5% CO_2_, 37°C and 95% humidity). Supernatant from PC3 and 3T3 cells was collected after 48 hrs from T75 flasks and directly transferred to 3D HS5 cells. HS5 cells were plated into 12 well plates on GFR Matrigel and left to adhere O/N in standard culture conditions before addition of PC3 and 3T3 conditioned media. Supernatant was replenished every 2 days. HS5 cells were imaged via Differential Inference Contrast (DIC) optics and processed for western analysis on days 3, 6 and 9 in culture.

### Live and fixed cell imaging

All fixed cells were imaged using either a PerkinElmer Opera™ Quadruple Excitation High Sensitivity Confocal Cell Imager with a PerkinElmer 20/.75 water iris, or an Olympus IX-81 Scanning Confocal microscope, with an Olympus PlanNeo-FLUAR 40/1 oil iris for multiple z-plane acquisition. Z-stacks of 160-180 z-planes with a step size of 0.4-0.8 μm were acquired with Olympus Fluoview Ver 1.7 b software (Olympus). 3D reconstructions of z-stacks were created in Imaris x64 Ver. 7 software (Bitplane Scientific Solutions). All live cell imaging was undertaken on the InCell 1000 (GE) Cell Imager using a GE 20/.75 air iris or on an Olympus Cell-R using an Olympus PlanNeo-FLUAR 20/.75 air iris. Images were compiled using Adobe Photoshop CS4 without further nonlinear digital manipulation.

### Quantification procedures and statistical analysis

Cell invasion by counting the number of migrated cells across 4 fields using ×20 magnification on the InCell 1000 (GE) and processed by an automated script generated by InCell Developer software (GE). Alternatively, quantification of the relative contribution of invaded PC3 and HS5 cells in co-culture was attained across 4 fields using ×40 magnification on an Olympus confocal and processed using Imaris x64 Ver. 7 software (Bitplane Scientific Solutions) volume and surface tool. Counts were averaged between 3 assay replicates. Densitometric analysis was performed using Image Lab software and expressed as a fold change in relation to loading controls and normalised against β-actin. This programme uses volume rendering which is a far more accurate measure of protein concentration as opposed to simple pixel intensity. Proliferation assays were quantified by KC4: Kineticalc for Windows (Version 3.4; Bio-Tek Instruments) and counts were averaged between 3 assay replicates. To quantify the relative contribution of proliferating PC3 and HS5 cells in co-culture and relative contribution of β1 and α6 expression, images were attained on the Olympus confocal (n = 10 per variable) and analysed using Imaris x64 Ver. 7 software volume and surface tool. Counts were averaged between 3 assay replicates. Statistical analysis was carried out using Graph-Pad Prism (Version 5) and statistical significance for all given variables was tested using Kruskal-Wallis test and Dunn’s Multiple Comparison test for post-hoc analysis.

## Abbreviations

PCa: Prostate cancer; EMT: Epithelial-to-mesenchymal transition; MET: Mesenchymal-to-epithelial transition; BPE: Bovine pituitary extract; EGF: Epidermal growth factor; ECM: Extracellular matrix; IF: Intermediate filament.

## Competing interests

There are no potential conflicts of interest or competing interests known to the authors of this paper.

## Authors’ contributions

LCW carried out the immunoassays, imaging, western blot, proliferation and invasion assays, conceived of the study, performed the statistical analysis, and designed and drafted the manuscript. TTG carried out the immunoassays, imaging, western blot assays and helped to draft the manuscript. VMA conceived of the study, and participated in its design and coordination and helped to draft the manuscript. All authors have read and approved the final manuscript.
